# IRS proteins and diabetic complications

**DOI:** 10.1007/s00125-016-4072-7

**Published:** 2016-08-11

**Authors:** Deborah P. Lavin, Morris F. White, Derek P. Brazil

**Affiliations:** 1 Centre for Experimental Medicine, School of Medicine, Dentistry and Biomedical Sciences, Queen’s University Belfast, 97 Lisburn Road, Belfast, BT9 7BL Northern Ireland UK; 2 Division of Endocrinology, Children’s Hospital Boston, Harvard Medical School, Boston, MA USA

**Keywords:** Diabetic complications, Eye, Heart, Insulin, Insulin receptor substrate, Kidney, Neuron, Review

## Abstract

IRS proteins are cellular adaptor molecules that mediate many of the key metabolic actions of insulin. When tyrosine is phosphorylated by the activated insulin receptor, IRS proteins recruit downstream effectors, such as phosphoinositide 3-kinase and mitogen-activated protein kinase, in order to elicit cellular responses such as glucose uptake, lipid metabolism and cell proliferation. There are two main IRS proteins in humans (IRS1 and IRS2), both of which are widely expressed. Given their central role in the insulin signalling pathway, it is not surprising that male mice lacking *Irs1* or *Irs2* present with elevated blood glucose or type 2 diabetes, respectively. For reasons yet to be identified, female *Irs2* ^−/−^ mice do not develop type 2 diabetes. A number of organs are affected by complications of diabetes; macrovascular complications include stroke and coronary artery disease, while nephropathy, neuropathy and retinopathy fall into the category of microvascular complications. Given the serious consequences of these complications on patient morbidity and mortality, it is essential to identify the molecular pathogenesis underlying diabetic complications, with a view to improving therapeutic intervention and patient outcomes. A number of recently published papers have converged on the hypothesis that the loss of insulin signalling and IRS proteins is instrumental to the development and/or progression of diabetic complications. This review will summarise some highlights from the published work in which this hypothesis is discussed.

**Table:** 

**Highlights**
• IRS proteins and cardiovascular disease
• IRS proteins and diabetic nephropathy
• IRS proteins in diabetic retinopathy
• IRS proteins in the brain
• IRS proteins in diabetic neuropathy

## Introduction

Insulin and IGF signalling require a family of scaffold proteins (IRS proteins) to integrate extracellular signals into intracellular responses, leading to cellular effects. There are two main IRS proteins in humans, IRS1 and IRS2, which are widely expressed in most human (and mammalian) tissues, whilst a third protein, IRS4, is mainly expressed in the hypothalamus [1]. All IRS proteins consist of an amino terminal pleckstrin homology (PH) domain and a phosphotyrosine binding (PTB) domain, followed by a long tail of tyrosine residues that act as phosphorylation sites to drive insulin/IGF-1 signalling [2]. IRS proteins can also be phosphorylated on Ser and Thr residues, and the majority of evidence suggests that this leads to reduced IRS protein expression and attenuated insulin signalling. Although similar in structure and sequence, the IRS proteins possess distinct roles in mammalian physiology. This is beautifully illustrated by the phenotypes of *Irs1*- vs *Irs2*-knockout mice. Male mice lacking *Irs1* have a small body size, and increased beta cell mass accompanied by mild metabolic defects [3, 4]. In contrast, male *Irs2* ^−/−^ mice are normal in size but develop diabetes at around 8 weeks, due to hepatic and peripheral insulin resistance and pancreatic beta cell insufficiency [3, 5]. The primary signalling pathway activated by IRS proteins is the phosphoinositide 3-kinase (PI3K)–protein kinase B (PKB/Akt) cascade, which regulates many downstream effectors such as glycogen synthase kinase 3β (GSK3β), mammalian target of rapamycin complex 1 (mTORC1) and mTORC2, and Forkhead transcription factors [2]. Changes in IRS protein function have been well studied in diabetes, in mouse models and human samples. This review will summarise a wealth of data describing the role of IRS proteins in diabetic complications, many of which may involve altered insulin and IGF-1 signalling as causal factors.

## IRS proteins and cardiovascular disease

Diabetes significantly increases the risks of cardiovascular disease (CVD). In the UK, CVD accounts for 48% of fatalities in people with either type 1 or type 2 diabetes [6, 7]. The risk of stroke, angina, myocardial infarction (MI) and heart failure is approximately double in diabetic patients. Intensive control of blood glucose has been shown to decrease the risk of non-fatal heart attack, stroke or death from CVD by 57% in diabetic patients [8]. Insulin has been proposed to mediate an anti-atherogenic effect on blood vessels via IRS–PI3K–Akt signalling [9]. This leads to activation of endothelial nitric oxide synthase (eNOS), expression of haemoxygenase-1 (HO-1) and vascular endothelial growth factor (VEGF) and decreased expression of vascular cell adhesion molecule-1 (VCAM-1), all of which are protective against vascular damage [9]. In contrast, insulin-mediated activation of growth factor receptor bound protein-2 / SH2-containing proto-oncogene / mitogen-activated protein kinase (MAPK) mediates a pro-atherogenic effect, increasing expression of endothelin-1 (ET-1) and plasminogen activator inhibitor-1 (PAI-1) and increasing the proliferation of contractile cells in the blood vessels [9]. In diabetes, which is characterised by an absence of insulin or increased peripheral insulin resistance (or both), a reduction in insulin receptor (IR) to IRS to PI3K to Akt signalling leads to acceleration of atherosclerosis because of the loss of anti-atherogenic insulin signalling [9]. Angiotensin II, NEFA and TNF-α contribute to this effect by increasing serine phosphorylation of IRS proteins, which leads to decreased Akt activation, reduced VEGF expression and poorer outcomes in CVD [9]. Protein kinase Cβ (PKCβ) isoforms such as PKCβ2 have been implicated in IRS2 phosphorylation on Ser303 and Ser675, leading to negative regulation of IRS2 tyrosine phosphorylation and reduced insulin signalling [10]. In addition, PKC inhibitors such as ruboxistaurin have been shown to reduce the severity of myocardial injury in rodent models of diabetic heart disease [11–13] and in a pig model of MI [14].

The essential role of insulin in healthy heart and blood vessel function has been highlighted by studies in which the IR was deleted from cardiac muscle and endothelial cells. Mice lacking IR expression in cardiac muscle (MIRKO) showed impaired cardiac performance at 6 months [15]. When these MIRKO mice had a single copy of *Igfr1* deleted, they died within 4 weeks of birth due to dilated cardiomyopathy [15]. Similarly, endothelial cell-specific *Insr*-knockout mice (EIRKO) displayed accelerated atherosclerosis when crossed with apolipoprotein E (ApoE) null mice [16]. Analysis of left ventricular myocardial biopsies from humans with type 1 diabetes showed a surprising increase in IR to IRS1 to PI3K to Akt signalling [17]. These changes were accompanied by a decrease in GLUT4 expression in the sarcolemma, suggesting defective myocardial insulin resistance in type 1 diabetes. This hypothesis is supported by data from the *ob*/*ob* model of type 2 diabetes, which, along with other data from animal models, is shown in Table 1.

**Table 1:** Summary of data linking IRS proteins to cardiac dysfunction

IRS involved	Mouse model	Organ affected	Main findings	Reference
IRS1, IRS2	Myocardium-specific *Irs1* ^−/−^; *Irs2* ^−/−^ mice	Heart	In the absence of both *Irs1* and *Irs2*, thinning walls, global dilatation, diastolic failure and decreased systolic activity were evident. Heart failure markers such as β-myosin heavy chain increased and interstitial fibrosis was associated with increased TGFβ1 and collagen 1 mRNA. Myocardium-specific *Irs1* ^−/−^; *Irs2* ^−/−^ mice died at 6–8 weeks of age, yet heterozygous myocardium *Irs1* ^+/−^; *Irs2* ^+/−^ mice survived to at least 6 months of age before fatal cardiac dysfunction developed	Qi et al (2013) [78]
	Mice with specific hepatic deletion of *Irs1* and *Irs2*	Liver	Hepatic deletion of *Irs1* and *Irs2* caused a decrease in cardiac IRS1 and IRS2, and similar, albeit less severe, effects on the heart were observed. These mice showed p38MAPK activation, which mediated IRS protein degradation in response to chronic insulin stimulation. These data suggest a new link between hepatic insulin signalling and IRS protein levels in the heart	Qi et al (2013) [78]
IRS1	*ob*/*ob* mice (model of type 2 diabetes)	Heart	Myocardial IRS1–PI3K activity was significantly increased in *ob*/*ob* mice vs control. These data were similar to IRS1–PI3K activity in both individuals with type 2 diabetes and those with left ventricular dysfunction, whose myocardium was insulin resistant. IR-β, IRS1 and total and phospho-Akt were also increased in *ob*/*ob* mice; however, total IRS1 was unchanged in individuals with type 2 diabetes and left ventricular dysfunction. Insulin-induced Akt phosphorylation was greater in control mice than in *ob*/*ob* mice with no alterations in total Akt	Cook et al (2010) [17]^a^

^a^Mouse- and human-based research summarised

### Data from human studies

Several epidemiological studies have identified polymorphisms that implicate IRS proteins in the incidence of diabetic complications. A specific Gly(972)Arg substitution mutation in the *IRS1* gene is a significant independent predictor of coronary artery disease (CAD) [18]. *IRS1* Gly(972)Arg mutations were detected in 6.8% of control and 18.9% of individuals with coronary atherosclerosis identified by angiography [18]. The *IRS1* Gly(972)Arg mutation was associated with an increased risk of CAD, independent of other factors such as smoking or hypertension, with an additional increase in risk in obese individuals or those with insulin resistance syndrome [18]. Diabetes incidence and plasma triacylglycerol levels are increased in CAD Gly(972)Arg mutation carriers compared with non-carriers [18]. Interestingly, *IRS1* Gly(972)Arg mutations were observed in 5.8% of the general population and 10.7% of individuals with type 2 diabetes, suggesting a genetic basis for increased CAD in the diabetic population [19].

A meta-analysis of 27 studies, carried out by Jellema et al, showed that *IRS1* Gly(972)Arg carriers had a 25% greater risk of developing type 2 diabetes [20]. Liu et al showed that the *IRS1* Gly(972)Arg mutation associates with serum ACE2 levels in patients following acute MI [21]. ACE2 was significantly elevated in acute MI patients vs control; however *IRS1* Gly(972)Arg carriers with acute MI had significantly decreased levels of ACE2, presenting with a more severe MI and a poorer prognosis [21]. As ACE2 negatively regulates the renin–angiotensin system, it has a cardioprotective role following its activation post MI and counteracts the adverse cardiac renin-angiotensin system effects [21]. In the presence of insulin, and under hypoxic conditions in vitro, *IRS1* Gly(972)Arg inhibited ACE2 expression in human cardiomyocytes, revealing a possible link between insulin and IRS protein signalling and cardiac renin–angiotensin system activation [21]. Another case–control study has supporting data from subjects of Punjabi origin, where *IRS1* Gly(972)Arg carriers or Arg homozygous individuals had a higher risk of CAD in the overall population [22]. However, contradictory data from a case–control study by Strohmer et al showed no association between the Gly(972)Arg mutation and CAD in male or female patients, or in obese patients, in the Austrian population, although the power of this study required to determine a causal role was suboptimal [23]. Data from Vats et al have shown that a single-nucleotide polymorphism (SNP) in *IRS2*—Gly(1057)Asp—is also associated with CAD, where Gly(1057)Asp carriers or Asp1057 homozygous individuals have an increased risk of developing CAD in the overall population of Punjabi origin, and in obese individuals [22]. Chan et al have shown that in the Taiwanese population, *IRS2* Gly1057 homozygous/heterozygous individuals are at increased risk of developing CAD, whereas Asp/Asp1057 individuals are protected against CAD [24]. However, further confirmation is needed due to some limitations of the study [24].

Diminished insulin to IRS signalling has been implicated in the poor prognosis of diabetic patients following a stroke [25]. Similar to the heart, insulin mediates a protective effect on cerebral blood vessels, and insulin resistance is implicated in increased stroke incidence in diabetes. Tight control of glucose reduces the risk of non-fatal stroke by 57% [8, 26], via similar protective mechanisms to those discussed above. IRS proteins may play a role in the response of neurons to ischaemia, as in a mouse model of obesity-induced type 2 diabetes there was reduced IRS to PI3K to Akt signalling in the brain [27]. In a recent study of type 2 diabetes patients, Zhang et al found that polymorphisms in *IRS1* were associated with higher platelet activity and suboptimal responses to drugs such as clopidogrel (Plavix), which is used to prevent platelet adhesion in patients at risk of stroke [28]. A summary of IRS signalling in the brain is provided later in this review.

## IRS proteins and diabetic nephropathy

Diabetic nephropathy is the leading cause of end-stage renal disease (ESRD) worldwide. Up to 40% of individuals with type 1 or type 2 diabetes will have clinically evident kidney disease during their lifetime [29]. The annual cost of chronic kidney disease to the National Health Service in England is estimated to be £1.45 billion [30]. Current treatment of diabetic nephropathy relies on tight glycaemic control, together with improved control of systemic and intraglomerular hypertension, using ACE inhibitors or angiotensin II receptor blockers as the mainstays of therapy [29]. Insulin signalling plays a critical role in kidney cell physiology and in the maintenance of the glomerular basement membrane (GBM). Defects in insulin signalling have been implicated in diabetic nephropathy at the level of the IR and IRS proteins (Table 2). The glomerular podocyte, the interdigitated foot processes of which form part of the GBM, responds to insulin by increasing glucose uptake via GLUT1 and GLUT4 transporters [31]. Nephrin is needed for this response, as it facilitates translocation of GLUT proteins to the plasma membrane via synaptobrevin (VAMP-2) binding [32]. The significance of these data for glomerular function in vivo was highlighted by the generation of podocyte-specific *Insr*-knockout (PodIRKO) mice [33]. At 8 weeks of age, PodIRKO mice developed podocyte foot effacement, apoptosis and albuminuria, accompanied by increased type IV collagen staining and glomerulosclerosis [33]. The likely mechanism for this diabetic nephropathy-like phenotype in PodIRKO mice was reduced VEGF-A production, which is stimulated by insulin in podocytes in vitro and in vivo [34]. These data tally with previous reports detailing defective filtration barrier development and diabetic nephropathy-like symptoms in mice lacking VEGF-A in podocytes [35]. Given the critical role of insulin signalling pathways in podocyte and GBM function, it is perhaps not surprising that numerous reports have also identified roles for IRS proteins in a range of kidney cells from mouse models. For example, IRS2 expression is enriched in glomerular podocytes, and podocytes from *Irs2* ^−/−^ mice are significantly insulin resistant, suggesting a dominance of IRS2 over IRS1 in these cells [36]. IRS2 is also overexpressed in kidney tubular epithelial cells in biopsies from humans with diabetic nephropathy [37]. More data supporting a role for IRS protein in diabetic kidney are summarised in Table 2.

**Table 2:** Summary of data describing roles for IRS proteins in kidney function and diabetic nephropathy

IRS protein involved	Rodent genotype/phenotype	Kidney region/cell affected	Main findings	Reference
IRS1	OLETF rats that developed diabetes and overt nephropathy	Renal cortex	IRS1 protein expression significantly reduced vs control LETO rats	Nakamura et al (2015) [79]
IRS1	ROP mice	Isolated mesangial cells	High glucose increased total IRS1 protein expression and IRS1 phosphorylation in mesangial cells from glomerulosclerosis-prone ROP mice, and levels of IGF-1 receptor and IGF-1 also increased. This is consistent with previous data implicating IRS1 as the main IRS protein involved in IGF-1 signalling. ROP mesangial cells expressed less IGFBP-2 vs control mice, and individuals with DN presented with reduced IGFBP-2 in glomeruli vs control individuals, indicating the potential for IGFBP-2 as a marker identifying patient susceptibility to DN. Low levels of IGFBP-2 may play a partial role in increased IGF-1 signalling via IRS1, as treatment of ROP mesangial cells with exogenous IGFBP-2 reduced glucose-induced increases in IRS1 phosphorylation, protecting cells from further damage	Fornoni et al (2006) [80]^a^; Myers et al (1993) [81]
IRS1	NOD mice	Isolated mesangial cells	Mesangial cells from NOD mice (model of type 1 diabetes) with nephropathy displayed phenotypic changes such as IGF-1 signalling pathway activation	Fornoni et al (2006) [80]
IRS1	*db*/*db* mice	Isolated mesangial cells	Mesangial cells from *db*/*db* mice (model of type 2 diabetes) also displayed phenotypic changes, such as IGF-1 signalling pathway activation	Fornoni et al (2006) [80]
IRS1	C57BL/6 mice	Isolated mesangial cells	High glucose treatment of control mesangial cells from glomerulosclerosis-resistant C57BL/6 mice yielded changes that were similar to those observed with mesangial cells from NOD and *db*/*db* mice. These data suggest that both type 1 and type 2 diabetes stimulate the IGF-1 pathway	Fornoni et al (2006) [80]
IRS2	Wild-type, *Irs1* ^−/−^ and *Irs2* ^−/−^ mice	Proximal tubule epithelial cells	Insulin-induced increases in tubular bicarbonate ion absorption and Akt phosphorylation were seen in wild-type mice, and this effect was decreased in *Irs2* ^−/−^ mice. No defects in bicarbonate absorption were seen in *Irs1* ^−/−^ mice, suggesting that IRS2 coordinates the effects of insulin upon tubular epithelial cell bicarbonate transport	Zheng et al (2005) [82]
IRS2	Wild-type mice	Tubular epithelial cells	IRS2 expression detected in embryonic kidney tubules, adult proximal and distal tubules and cortical collecting duct. BMP-7 increased IRS2 signalling in HK-2 proximal tubule epithelial cells	Hookham et al (2013) [37]
IRS2	*Irs2* ^−/−^ mice	Podocytes	*Irs2* ^−/−^ podocytes were found to be insulin resistant with respect to Akt signalling and GLUT4-mediated glucose uptake in response to insulin	Santamaria et al (2015) [36]

^a^Mouse- and human-based research summarised

DN, Diabetic nephropathy; IGFBP-2, IGF binding protein-2; LETO, Long–Evans Tokushima Otsuka (rats); OLETF, Otsuka Long–Evans Tokushima fatty (rats); ROP, ragged olygosyndactilism pintail (mice)

### Epidemiology data on IRS proteins in diabetic nephropathy

Decreasing GFR is widely accepted as a hallmark of diabetic nephropathy, where an inverse relationship exists between GFR and the progressive stages of diabetic nephropathy [38]. A region on chromosome 2q35-37 was highlighted in a cohort of Mexican-American diabetes patients as an area with the strongest GFR linkage [39]. Within this region, *IRS1* was identified as a potential candidate gene [39]. Of note, this *IRS1* mutation is also associated with increased risk of CAD (see above). *IRS1* SNPs were identified and analysed in Mexican-American individuals, to identify links between declining GFR and type 2 diabetes [39]. A specific missense mutation in *IRS1* occurring at codon 972 (Gly[972]Arg) was found to be the only SNP out of 18 that conferred association with GFR and serum triacylglycerol levels [39]. This SNP was responsible for 26% of the GFR–chromosome 2q linkage, with carriers of this mutation presenting with significantly lower GFR [39]. Consistently, human mesangial cells transfected with mutant Gly(972)Arg *IRS1* displayed lower insulin-induced phosphorylation of IRS1 and Akt [39]. These data suggest that even a single mutated codon in *IRS1* can negatively influence insulin signalling and an individual’s GFR and renal function.

Chromosome 13q33.3 has also been implicated as a diabetic nephropathy susceptibility locus in patients with type 1 or type 2 diabetes [40]. This particular locus is situated at an intergenic region close to both *MYO16* (encoding myosin heavy chain Myr 8) and *IRS2* [40], which is located in region 13q34 [41]. Following a genome-wide association scan of the Genetics of Kidneys in Diabetes (GoKinD) collections, 13q33.3 was one of four loci associated with diabetic nephropathy in patients with type 1 diabetes [40]. In a Japanese subset of patients with type 2 diabetes, SNPs in these same loci increased diabetic nephropathy risk [40]. In the Joslin Study of Genetics of Nephrology in Type 2 Diabetes collection, multiple SNPs on chromosome 13q33.3 were identified with one particular SNP (rs1411766) exclusively associated with risk of early diabetic nephropathy and a trend towards an association with diabetic nephropathy in type 2 diabetes [40]. These data suggest that inherited changes in or around the *IRS2* gene may contribute to the genetic predisposition or increased risk of developing diabetic nephropathy in some diabetic patients.

### IRS proteins in diabetic nephropathy: a novel therapeutic target?

Given the changes in IRS proteins in the diabetic kidney, the question arises: can we alter IRS protein levels to provide benefit and reduce the severity of diabetic nephropathy in patients? A second question arises: would increasing or decreasing IRS protein levels (and therefore insulin signalling) be of benefit in the diabetic kidney? Data from the Coward group showed that insulin-mediated VEGF production is critical for podocyte survival in the kidney, suggesting that increasing IRS protein levels may help to rescue insulin signalling in the diabetic kidney [33]. However, treatment of *db*/*db* mice with bioactive peptides nephrilin or anephril significantly reduced IRS2, p-IRS1-Ser307, serum/glucocorticoid-regulated kinase 1 and phospho-PKC-α/β which, when elevated, are associated with diabetic nephropathy [42]. A concomitant decrease in albuminuria was also detected in these mice, suggesting a beneficial effect of these peptides on renal function [41]. Streptozotocin (STZ)-treated mice yielded higher nuclear expression of IRS2, Rictor and phospho-PKC in fractionated renal tissue extracts, which was reduced by nephrilin in vivo [42]. These data suggest that, as a component of mTORC2, a nuclear complex of IRS2 and Rictor exists that is disrupted by nephrilin, potentially implicating this complex in albuminuria in diabetic nephropathy [42]. In contrast, data from Wei et al suggest that modulation of hypoxia-mediated regulation of IRS2 in the liver can sensitise hepatic insulin signalling [43]. VEGF inhibition improved glucose tolerance in *db*/*db* mice and this augmented insulin signalling via IRS2 induction. Hepatic hypoxia was induced, which subsequently activated hepatic hypoxia-inducible factor-2α (HIF-2α) and improved insulin signalling as HIF-2α promotes IRS2 expression [43]. Complementary work from Taniguchi et al showed that specific hepatic deletion of the gene encoding prolyl hydroxylase 3 (*Phd3*, also known as *Egln1*) in mice modified insulin sensitivity by eliciting HIF-2α stabilisation, resulting in greater IRS2 transcription and downstream Akt signalling [44]. The relationship between HIF-2α and IRS2 therefore provides a new therapeutic approach to increase IRS2 levels in the treatment of diabetes and related complications, should this be demonstrated to be therapeutically advantageous in patients [44]. We and others are currently exploring the effects of increasing IRS2 levels in the diabetic kidney using similar hypoxia-mediated strategies.

## IRS proteins in diabetic retinopathy

Diabetic retinopathy is a common microvascular complication of diabetes characterised by damage to a number of retinal cell types including retinal pigment epithelia, Müller cells and retinal vascular cells [45]. A significant feature of diabetic retinopathy is the loss of retinal blood vessels, leading to retinal ischaemia/hypoxia and then to pathogenic neovascularisation and progressive vision loss [46]. IRS proteins are expressed in the normal retina, and both IRS1 and IRS2 have been implicated in normal retinal development and diabetic retinopathy. Some of these data are discussed below.

### Retinal Müller cells: a primary site of IRS signalling in the retina

IRS proteins are heavily phosphorylated on tyrosine, serine and threonine residues [37, 47, 48]. Aguirre et al originally showed that c-Jun N-terminal kinase (JNK)-induced IRS1^Ser307^ phosphorylation prevents IR–IRS interactions, resulting in impaired insulin signalling [49, 50]. In contrast, Copps et al showed that substitution of Ser307 with Ala resulted in severe insulin resistance and impaired insulin signalling, thus positively implicating Ser307 in maintaining insulin signalling and sensitivity [27]. With regard to the retina, the Steinle group showed in both rat retinal Müller cells and human retinal endothelial cells grown in high glucose, that TNF-α increased IRS1^Ser307^ phosphorylation, insulin resistance and retinal cell death [51–53]. This effect was reversed by the β-adrenergic agonist salmeterol, suggesting that β-adrenergic receptor agonists can restore insulin signalling and protect retinal Müller cells from apoptosis in diabetes [53]. Similar effects were seen in retinal endothelial cells, where hyperglycaemia caused elevated TNF-α, increased IRS1^Ser307^ phosphorylation and loss of IRS1 protein, likely via suppressor of cytokine signalling 3 (SOCS3)-mediated targeting to the proteasome [54]. Given the critical role of retinal capillary loss during diabetic retinopathy pathogenesis, these data provide evidence that rescuing insulin signalling may decrease retinal endothelial cell apoptosis, thus potentially improving diabetic retinopathy outcomes. Supporting this hypothesis, two reports indicated that pioglitazone, a peroxisome proliferator-activated receptor-γ (PPARγ) agonist reduced TNF-α-mediated IRS1^Ser307^ phosphorylation and insulin resistance in diabetic rat retinas, with a concomitant improvement in retinal function [55]. Pioglitazone was shown to decrease Müller cell and retinal endothelial cell apoptosis, likely via increased insulin-mediated pro-survival signalling [55]. Similar data were obtained using sodium salicylate [56], which was shown to reverse insulin resistance via inhibition of TNF-α-mediated Ikkβ-driven inflammatory responses [57, 58].

Mouse models have also demonstrated key roles for IRS proteins in retinal development as well as disease. A summary of these data is shown in Table 3.

**Table 3:** Summary of data linking IRS proteins to diabetic retinopathy

IRS involved	Rodent genotype/phenotype	Organ affected	Main findings	Reference
IRS2	*Irs2* ^−/−^ mice	Eye (retina)	*Irs2* gene deletion triggered photoreceptor apoptosis with a 50% reduction in cells by day 14, with almost complete photoreceptor loss by 16 months of age. In contrast, normal retinal morphology was maintained in *Irs1* ^−/−^ mice at up to 2 years of age. Retinas from *Irs2* ^−/−^ mice displayed higher caspase-3 cleavage and activation, and lower Akt phosphorylation, consistent with apoptotic cell death. Expression of rhodopsin (the light-sensing G-protein-coupled receptor in the retina) was reduced in *Irs2* ^−/−^ retinas and retinal electrical function showed that *Irs2* ^−/−^ mice had reduced dark- and light-adapted responses, suggesting lower rod and cone activity, respectively	Yi et al (2005) [45]
IRS2	9- and 12-week-old *Irs2* ^−/−^ mice	Eye (retina)	At 9 weeks, activation of Müller glial cells was apparent, as demonstrated by outer retinal layers that were considerably thinner than their wild-type counterparts. Thinning extended to each retinal layer in 12-week-old *Irs2* ^−/−^ mice. Changes in microglia were also detected at 9 weeks with swelling/retraction of microglial processes observed. In both age groups, shortened rod segments were apparent, and rhodopsin distribution in the rod photoreceptors of the outer nuclear layer was abnormal. Photoreceptor atrophy, as well as synaptic changes at the outer plexiform layer were also observed. Retinal ganglion cell degeneration was present in 12-week-old *Irs2* ^−/−^ mice with the progressive loss of these cells observed	Albert-Fort et al (2014) [83]; Kern and Barber (2008) [84]
IRS1, IRS2	STZ-induced model of type 1 diabetes in Sprague–Dawley rats	Eye (retina)	At 1 month post STZ-induced diabetes, retinal IRS1/2-associated PI3K activity, Akt1 and Akt3 kinase activity, and p70S6K/pGSK3β were decreased with no change in retinal IRS1/2 expression or tyrosine phosphorylation. Such rapid signalling changes and alterations in p70S6K and GSK3β as a result of hyperglycaemia provide further evidence of a role for insulin signalling in retinal cell survival. After 3 months, IR-β expression, autophosphorylation and kinase activity was decreased in diabetic retina. Moreover, IRS2 protein expression was decreased whereas IRS1 was unchanged. These data suggest that IR signalling is compromised relatively early in the diabetic retina, and suggests a critical role for reduced insulin in IRS2 signalling in progressive retinal degeneration	Reiter et al (2006) [85]

P706K, phospho-p70 S6 kinase

## IRS proteins in the brain

The main diabetic complication affecting the brain is the increased risk of stroke (discussed above). However, defective insulin signalling has also been identified in the central nervous system in diabetes, and has been linked to altered nutrient homeostasis, an increased risk of Alzheimer’s disease and changes in lifespan. The role of insulin signalling in the brain has been reviewed expertly elsewhere [59]. The main effects of insulin in the brain appear to be on energy homeostasis, neuronal proliferation, Tau phosphorylation and reproductive endocrinology [59]. Centrally administered insulin can improve peripheral insulin sensitivity in the liver by reducing hepatic glucose production [60]. Mice lacking the IR in neurons (NIRKO mice) developed diet-sensitive obesity, with increased body fat and plasma leptin levels [61]. Female mice lacking IRS2 are infertile, due to reduced levels of luteinising hormone, prolactin and sex steroids, linking insulin signalling to hypothalamic control of reproductive endocrinology [62]. IRS2 expression in the hypothalamus is also important in energy homeostasis, as mice lacking IRS2 in a specific subset of hypothalamic neurons display hyperphagia, obesity and increased body length [63]. Whole-body *Irs2* ^−/−^ mice have brains that are 30% smaller than wild-types, due to a defect in neuronal proliferation during development [64]. Elevated Tau phosphorylation was also detected in *Irs2* ^−/−^ mouse brains, suggesting a potential link between IRS2 and Alzheimer’s disease [64]. Consistently, levels of IRS2 (and IRS1) were reduced in the neurons of the temporal cortex of post-mortem human brains from Alzheimer’s patients compared with controls [65]. Others have demonstrated post-mortem insulin resistance in the brains of Alzheimer’s patients, specifically in the hippocampus, providing a link to cognitive defects and decreased memory formation [66]. Mice lacking IRS2 demonstrated deficiencies in *N*-methyl-d-aspartate receptor-mediated long-term potentiation and hippocampal plasticity [67, 68]. Increased serine phosphorylation of IRS proteins, a feature of insulin resistance, was also detected in the brains of Alzheimer’s patients [66]. Co-localisation of pSer-IRS1 and amyloid-β was demonstrated in these brains, and amyloid-β oligomers injected directly into the brain stimulated IRS1 protein phosphorylation and JNK activation [69]. Importantly, exendin-4, a glucose-lowering drug that activates glucagon-like peptide-1 (GLP-1) signalling, was shown to prevent this amyloid-β-induced insulin resistance and increase spatial memory in non-diabetic mice [69]. Together, these data support the notion that changes in insulin signalling during Alzheimer’s disease may represent an insulin-resistant state in the brain, and that targeting these changes may improve outcomes for Alzheimer’s patients [66].

## IRS proteins in diabetic neuropathy

Diabetic neuropathy is a common complication of diabetes, affecting 50% of patients. Many patients suffer from nerve pain, reduced proprioception (the body’s ability to sense joint movement and position) and peripheral insensitivity, leading to foot ulcers that may in severe cases require amputation [70]. Many different pathways have been implicated in the pathogenesis of diabetic neuropathy, including decreased neurotrophic growth factors such as IGF-1 [71], advanced glycation end-products and oxidative stress [72]. Impaired insulin signalling has been implicated in the pathogenesis of diabetic neuropathy. Insulin is a neurotrophic factor for the dorsal root ganglion, and IRS proteins are expressed in these cells, with IRS2 expression being most abundant in both myelinated and unmyelinated neurons [70]. In animal models of diabetes, several reports have identified a reduction in insulin sensitivity of dorsal root ganglion cells (summarised in Table 4). Reduced IRS2 expression was linked to hyperalgesia in diabetic mice, which was reversed by inhibition of the JAK2/signal transducer and activator of transcription 3 (STAT3) pathway [73]. Importantly, incretin drugs that are widely used to treat type 2 diabetes have been shown to reduce the severity of diabetic neuropathy in pre-clinical models. Exendin-4, vildagliptin and alogliptin all improved nerve conductance velocity in diabetic rats and reversed nerve damage [74–76]. These effects were linked to an improvement in both GLP-1 and insulin signalling, with expression of IRS2 being increased in neurons from diabetic animals treated with vildagliptin [77]. Thus, the preservation or restoration of neuronal insulin signal transduction pathways and IRS2 expression would seem to be a useful avenue for exploring new and improved therapeutic interventions in diabetic neuropathy.

**Table 4:** Summary of animal model data implicating IRS proteins in diabetic neuropathy

IRS involved	Rodent genotype/phenotype	Organ affected	Main finding(s)	Reference
IR, IRS1	*ob*/*ob* mice (model of type 2 diabetes)	Spinal dorsal horn region of the spinal cord	Diabetic *ob*/*ob* mice displayed PDN in the form of mechanical hyperalgesia, as demonstrated by low paw-withdrawal thresholds. This decrease in withdrawal threshold coincided with reduced p-IRS1 and IR-positive spinal dorsal horn neurons in these mice. As PDN progresses, IR and p-IRS1 levels decline. Also, with progression of PDN, pJAK2 and p-STAT3 levels increase, with specific activation of JAK2/STAT3 occurring in this area. Administration of a JAK2/STAT3 pathway inhibitor reverts the pain threshold to normal levels in *ob*/*ob* mice. The JAK/STAT inhibitor also prevented the decrease in IR and p-IRS1, suggesting a link between JAK/STAT, IR/IRS1 signalling and PDN	Kou et al (2013) [73]
IRS2	Diabetic GK rats	Neurons	Oral administration of vildagliptin reduced neuronal atrophy and improved neuropathy as evaluated by motor and sensory nerve conduction velocity. Vildagliptin preserved intra-epidermal nerve fibre density in GK rats, which indicates beneficial neuropathic effects. Vildagliptin increased GLP-1 signals such as CREB; PKB/Akt phosphorylation in the dorsal root ganglion, with changes in IRS2^Tyr/^IRS2^Ser^ phosphorylation also seen. Crosstalk exists between insulin and GLP-1 at least in part through CREB phosphorylation, which promotes IRS2 expression. Of note, hyperglycaemia in these diabetic GK rats was unchanged following vildagliptin treatment, suggesting that vildagliptin functions to inhibit neuropathy development independently of hyperglycaemia	Tsuboi et al (2015) [77]
IR-β, IRS1, IRS2	ZDF rats, a long-term model of PDN	Sensory neurons and dorsal root ganglion	Progressive slowing of nerve conduction in the ZDF rats is detected. ZDF rats are more sensitive to mechanical withdrawal (similar to *ob*/*ob* mice), suggesting pain sensation from a non-painful stimulation of the skin. Dorsal root ganglion mRNA levels of IR-β, IRS1 and IRS2, Akt, PI3K and GLUT4 were preserved or slightly increased in ZDF rats	Brussee et al (2008) [86]
IRS2	*ob*/*ob* mice (model of type 2 diabetes), and STZ C57BL/6 mice (model of type 1 diabetes)	Dorsal root ganglion	Total IRS2 levels decreased in diabetic *ob*/*ob* dorsal root ganglion neurons. IRS2 Ser phosphorylation increases in dorsal root ganglion neurons from diabetic *ob*/*ob* mice and dorsal root ganglion neurons from an STZ mouse model. Suboptimal dorsal root ganglion insulin signalling may be caused by increased IRS serine phosphorylation, leading to IRS degradation, decreasing IR/IRS interaction. In healthy neurons, insulin increases neurite outgrowth. Neurons from *ob*/*ob* mice were unable to efficiently respond to insulin. Increased IRS2 serine phosphorylation and subsequent insulin resistance may modulate neuronal insulin signalling to promote peripheral nerve dysfunction leading to PDN.	Aguirre et al (2000) [49]; Grote et al (2011) [70]; Rui et al (2001) [87]

CREB, cAMP response element binding protein; GK, Goto–Kakizaki (rats); PDN, painful diabetic neuropathy; ZDF, Zucker diabetic fatty (rats)

## Concluding remarks

**Fig. 1:**
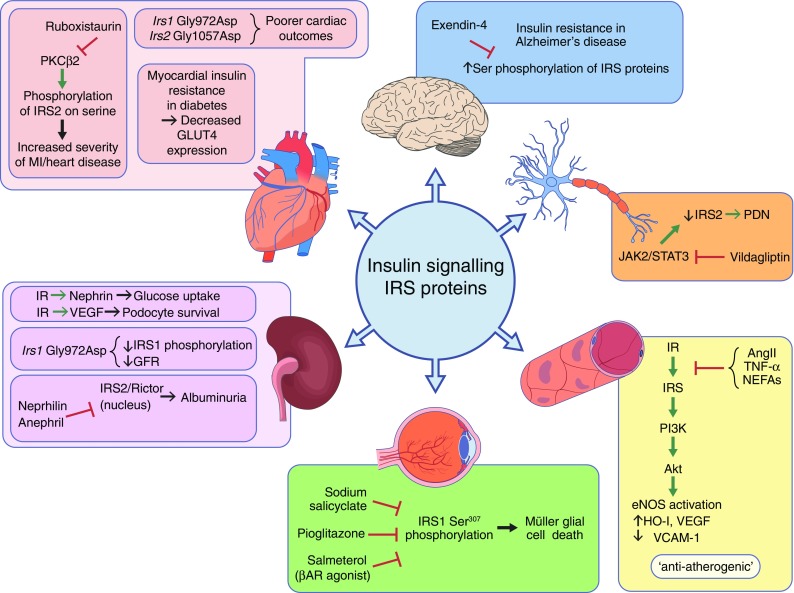
IRS proteins in organs affected by diabetic complications. A selection of data from animal and human studies supporting changes in IRS proteins during diabetic heart disease (pink panel), diabetic nephropathy (purple panel), diabetic retinopathy (green panel), diabetic vascular disease (yellow panel), diabetic neuropathy (orange panel) and Alzheimer’s disease (blue panel). Details and supporting references are provided in the text. Red T-bar represents inhibition; green arrow represents activation; black arrow represents ‘leading to’. AngII, angiotensin II; βAR, β-adrenergic receptor; PDN, painful diabetic neuropathy

A summary of the role of IRS proteins in organs affected by diabetic complications is shown in Fig. 1. It is clear from the literature that reduced insulin signalling is implicated not just in the aetiology of diabetes but also in the pathogenesis of diabetic complications. Given their central role in insulin signalling, it is perhaps not surprising that abundant evidence exists to show that IRS proteins are implicated in vascular complications of diabetes. A slew of mouse data and genetic studies in human patients strengthen the argument that changes in IRS function contribute to diabetic complications in affected organs such as the heart, kidney, eye and nervous system. The vascular aetiology of many diabetic complications makes it reasonable to assume that changes in IRS protein expression or phosphorylation occur as a result of chronic hyperglycaemia, which triggers vascular damage. Data from mice showing that increasing IRS2 levels provides relief in terms of hepatic insulin resistance are very encouraging. Emerging gene-editing technologies, such as CRISPR (clustered regularly interspaced short palindromic repeats)/Cas9 and TALENS (transcription activator-like effector nucleases), will allow deletion or insertion of IRS1, IRS2 or both proteins in vascular cells damaged in diabetes. These future experiments will shed new light on the role of IRS proteins in diabetic vascular complications, and also expand our ability to provide novel therapeutic strategies aimed at boosting IRS protein levels and improving the outcomes for patients with diabetic complications.

## Abbreviations

ApoE: Apolipoprotein E
CAD: Coronary artery disease
CVD: Cardiovascular disease
ESRD: End-stage renal disease
eNOS: Endothelial nitric oxide synthase
ET-1: Endothelin-1
GBM: Glomerular basement membrane
GLP-1: Glucagon-like peptide-1
GSK3β: Glycogen synthase kinase 3 β
HIF-2α: Hypoxia-inducible factor-2α
HO-1: Haemoxygenase-1
IGF-1R: IGF-1 receptor
IGFBP-2: IGF binding protein 2
IR: Insulin receptor
Ikkβ: Inhibitor of nuclear factor κ-B kinase subunit β
JAK2: Janus kinase 2
JNK: c-Jun N-terminal kinase
MI: Myocardial infarction
mTORC: Mammalian target of rapamycin complex
MAPK: Mitogen-activated protein kinase
PAI-1: Plasminogen activator inhibitor-1
PH: Pleckstrin homology
PI3K: Phosphoinositide 3-kinase
PKB: Protein kinase B/Akt
PKCβ: Protein kinase Cβ
PodIRKO: Podocyte-specific *Insr*-knockout
PPARγ: Peroxisome proliferator-activated receptor-γ
PTB: Phosphotyrosine binding
SNP: Single-nucleotide polymorphism
STAT3: Signal transducer and activator of transcription 3
STZ: Streptozotocin
VCAM-1: Vascular cell adhesion molecule-1
VEGF: Vascular endothelial growth factor
